# Quantitative *Ex Vivo* MRI Changes due to Progressive Formalin Fixation in Whole Human Brain Specimens: Longitudinal Characterization of Diffusion, Relaxometry, and Myelin Water Fraction Measurements at 3T

**DOI:** 10.3389/fmed.2018.00031

**Published:** 2018-02-20

**Authors:** Anwar S. Shatil, Md Nasir Uddin, Kant M. Matsuda, Chase R. Figley

**Affiliations:** ^1^Biomedical Engineering Graduate Program, University of Manitoba, Winnipeg, MB, Canada; ^2^Neuroscience Research Program, Kleysen Institute for Advanced Medicine, Winnipeg Health Sciences Centre, Winnipeg, MB, Canada; ^3^Department of Radiology, University of Manitoba, Winnipeg, MB, Canada; ^4^Division of Diagnostic Imaging, Winnipeg Health Sciences Centre, Winnipeg, MB, Canada; ^5^Department of Pathology, University of Manitoba, Winnipeg, MB, Canada; ^6^Department of Psychological and Brain Sciences, Johns Hopkins University, Baltimore, MD, United States

**Keywords:** *ex vivo*, fixation, formalin, longitudinal, postmortem, human brain, MRI, myelin, diffusion, T_1_, T_2_

## Abstract

**Purpose:**

Postmortem MRI can be used to reveal important pathologies and establish radiology–pathology correlations. However, quantitative MRI values are altered by tissue fixation. Therefore, the purpose of this study was to investigate time-dependent effects of formalin fixation on MRI relaxometry (T_1_ and T_2_), diffusion tensor imaging (fractional anisotropy, FA; and mean diffusivity, MD), and myelin water fraction (MWF) measurements throughout intact human brain specimens.

**Methods:**

Two whole, neurologically-healthy human brains were immersed in 10% formalin solution and scanned at 13 time points between 0 and 1,032 h. Whole-brain maps of longitudinal (T_1_) and transverse (T_2_) relaxation times, FA, MD, and MWF were generated at each time point to illustrate spatiotemporal changes, and region-of-interest analyses were then performed in eight brain structures to quantify temporal changes with progressive fixation.

**Results:**

Although neither of the diffusion measures (FA nor MD) showed significant changes as a function of formalin fixation time, both T_1_ and T_2_-relaxation times significantly decreased, and MWF estimates significantly increased with progressive fixation until (and likely beyond) our final measurements were taken at 1,032 h.

**Conclusion:**

These results suggest that T_1_-relaxation, T_2_-relaxation and MWF estimates must be performed quite early in the fixation process to avoid formalin-induced changes compared to *in vivo* values; and furthermore, that different *ex vivo* scans within an experiment must be acquired at consistent (albeit still early) fixation intervals to avoid fixative-related differences between samples. Conversely, *ex vivo* diffusion measures (FA and MD) appear to depend more on other factors (e.g., pulse sequence optimization, sample temperature, etc.).

## Introduction

Postmortem MRI has become a popular tool for assessing brain microstructure, structural development, and pathology with high resolution, and it has been increasingly used in combination with histological staining and proteomic approaches to probe underlying mechanisms of various quantitative imaging methods (1–5). From a biomedical imaging perspective, *ex vivo* MRI of postmortem tissue offers several benefits over *in vivo* scanning, including no participant or physiological (cardiac, respiratory, pulsatile, etc.) motion artifacts and no time restriction for data acquisition, which therefore allows images to be acquired with much higher spatial resolution and signal-to-noise ratio (SNR) (6–8). As a result, *ex vivo* MRI offers unprecedented potential to discover novel and interesting features of postmortem tissue architecture both in neurologically healthy and diseased brains by applying advanced MRI techniques, such as diffusion tensor imaging (DTI) (9), relaxometry methods (T_1_, T_2_, and T_2_*) (10–13), and multi-component T_2_-relaxation myelin water imaging (MWI) (14). For example, a small number of *ex vivo* studies have effectively combined MWI with histopathology, and validated a unique MR property—myelin water fraction (MWF)—as a marker of myelin integrity using tissue samples that were affected by multiple sclerosis (15–18). Thus, *ex vivo* MRI can offer valuable quantitative measures for validating *in vivo* research techniques and can also be used for a wide range of *ex vivo* applications (e.g., guiding targeted histological sampling and identifying microstructural pathologies or inconsistencies in postmortem brains that could otherwise be overlooked during macroscopic inspection in routine autopsy).

However, to prevent postmortem degradation, tissues are typically preserved with chemical fixatives—most commonly formaldehyde or formalin (19–21)—that are known to alter various MRI properties. For example, formalin fixation is known to shorten the T_1_- and T_2_-relaxation times of tissues compared to their *in vivo* state after a certain period (8, 22–24). While the exact mechanisms for these formalin-induced effects are still debatable, a few postmortem studies on non-human brains and small human brain tissue samples have measured these effects as a function of temperature (2, 25, 26), postmortem interval (i.e., the time elapsed between death and the start of fixation) (6, 27, 28), scan interval (i.e., the tissue fixation time) (8), MR parameters (29), or embedding media (30).

In order to better address some of the concerns related to postmortem human brain imaging, it is important to characterize the changes of different MRI properties in non-diseased human brains due to formalin fixation. Thus, in this work, we examine the quantitative changes of DTI-based fractional anisotropy (FA) and mean diffusivity (MD), relaxation times (T_1_, T_2_), and T_2_-relaxation based MWF measurements in a number of deep gray matter (GM) and white matter (WM) structures to explain the possible implications of progressive formalin fixation in whole human brain specimens. Although fixation-induced relaxation and diffusion changes have been previously investigated in human brain tissue, the current study is (to the best of our knowledge) the first characterization of MWF changes in whole formalin-fixed brains. Given that fixation has been shown to shorten T_2_-relaxation times, and MWF estimates are derived from short T_2_-relaxation water compartments, we hypothesize that MWF measurements will be artificially inflated as a function of tissue fixation. However, no previous studies have characterized this phenomenon. Therefore, by empirically measuring all of these signals over time (i.e., FA, MD, T_1_-relaxation, T_2_-relaxation, and MWF), our findings are expected to have important implications for future postmortem brain MRI studies by elucidating the time-course of *ex vivo* MRI signal changes in whole, fixed human brain samples.

## Materials and Methods

### Sample Preparation

This study was conducted with prior approval from the University of Manitoba Biomedical Research Ethics Board (BREB). Two human brain specimens—both female (Subject 1 = 71 years old; Subject 2 = 74 years old)—were obtained with prior consent. Each patient’s medical history was pre-screened by a neuropathologist to exclude cases with neurological disorders or medico-legal cases (i.e., those with unknown or unnatural causes of death). Both brains were subjected to a standard neuropathology examination following the study to ensure that incidental pathological findings were not identified.

Both volunteers for the study were palliative care patients who passed away in the hospital. Therefore, the times of death are well-established, and both bodies were transferred to the hospital morgue and refrigerated shortly after death (within 1–2 h). Each brain, including the dura mater, was then carefully removed as soon as possible (Subject 1 = 33 h total; Subject 2 = 55 h total), a MRI fiducial marker was attached to confirm the orientation of cerebral hemispheres in subsequent images, and the whole brain was immersed in 10% phosphate buffered formalin solution (Sigma-Aldrich Product ID: HT501128) inside a 3.8-L air- and liquid-tight MRI-compatible container using a previously reported *ex vivo* brain MRI protocol (31). For Subject 1, formalin was carefully injected into the posterior horn of the lateral ventricle using a needle syringe to eliminate a large air bubble that was identified during the initial scan, but this was not necessary for Subject 2 (as no trapped air bubbles were observed). Once immersed in formalin, the brain specimens were stored at room temperature (22 ± 1°C) between scans and, in accordance with standard neuropathology procedures at our site, the formalin was replaced approximately once per week (Table 1).

**Table 1:** Time of formalin change in hours after first immersion in the container.

Formalin change	Subject 1 (hours)	Subject 2 (hours)
1st	120	24
2nd	288	168
3rd	500	336
4th	672	500
5th	–	672

### Image Acquisition

Each brain specimen was scanned at room temperature (22 ± 1°C) at the same 13 time points (i.e., 0, 12, 24, 46, 120, 168, 211, 288, 336, 500, 672, 840, and 1,032 h) after the initiation of formalin fixation. All MRI scans were performed as previously described (31), in a whole-body 3T Siemens Magnetom Verio scanner (Siemens Healthcare, Erlangen, Germany) that was equipped with a 12-channel head coil and a 4-channel (knee) flex coil to acquire uniform signal throughout the field of view (FOV), and foam pads placed under and around the container to hold it in place and reduce scanner vibrations.

The total scan time for each MRI session was approximately 60 min. Diffusion measurements were performed by means of a single-shot fast 2D segmented spin echo, echo planar imaging sequence, that had the following scan parameters: 30 diffusion-encoded images [*b* = 700 s/mm^2^; maximum gradient amplitude = 45 mT/m; gradient duration (delta) = 20.2 ms; effective diffusion time (DELTA) = 25.7 ms], 5 reference images (*b* = 0 s/mm^2^), repetition time (TR) = 8,800 ms, echo time (TE) = 73.6 ms, flip angles = 90° (excitation) and 180° (refocusing), receiver bandwidth = 3,064 Hz/pixel, fat suppression = on, number of slices = 55, slice thickness = 2.5 mm, matrix size = 192 × 192, FOV = 240 mm × 240 mm, resolution = 1.25 mm × 1.25 mm × 2.5 mm, number of averages = 2, total scan time = 11 min. T_1_-relaxometry data were acquired using a 3D magnetization prepared rapid acquisition gradient echo (MPRAGE) sequence with two inversion times and two flip angles (MP2RAGE) (32).^1^ The parameters for this sequence were TR = 5,000 ms, TE = 2.87 ms, inversion times (TIs) = 700 and 2,500 ms (respectively), flip angles = 4° and 5° (respectively), receiver bandwidth = 240 Hz/pixel, fat suppression = off, matrix size = 128 × 140 × 176, FOV = 256 mm × 234 mm × 176 mm, resolution = 1.83 mm × 1.83 mm × 1 mm, and acquisition time = 11 min. Due to a temporary software license issue for the MP2RAGE sequence, T_1_-relaxometry data could not be acquired for the first four time points (i.e., 0, 12, 24, and 46 h scans) of Subject 1. As a result, conventional T_1_-weighted images were obtained using a conventional 3D MPRAGE sequence (33) in order to facilitate co-registration, spatial normalization, and image segmentation at these time points, which were then used to spatially normalize the DTI, T_2_-relaxation, and MWF maps (see below). The parameters for the conventional MPRAGE sequence were TR = 1,900 ms, TE = 2.49 ms, TI = 900 ms, flip angle = 9°, receiver bandwidth = 170 Hz/pixel, fat suppression = off, matrix size = 512 × 512 × 176, resolution = 0.49 mm × 0.49 mm × 0.98 mm, FOV = 250 mm × 250 mm × 172 mm, and scan time = 5 min. A multi-echo 3D combined gradient and spin echo (GRASE) sequence was used to acquire images with 32 different echo times (34) and was used for subsequent T_2_-relaxometry and multi-component MWI measurements (35). Scan parameters for the 3D GRASE sequence were: TR = 1,030 ms, first TE (TE1) = 10 ms, echo train length = 32, echo spacing = 10 ms, flip angles = 90° (excitation) and 180° (refocusing), receiver bandwidth = 1,250 Hz/pixel, fat suppression = on, matrix size = 160 × 120 × 22, FOV = 240 mm × 180 mm × 110 mm, resolution = 1.25 mm × 1.25 mm × 5 mm, acquisition time = 15 min.

Baseline (i.e., time = 0 h) SNR calculations were then performed on mid-axial 2D images obtained from each pulse sequence. This was achieved by dividing the average signal intensity within the brain by the SD of the noise in air outside of the bucket. The resulting SNR values for each pulse sequence are shown for Subject 2 in Table 2.

**Table 2:** Signal-to-noise ratio (SNR) measurements at first time point from Subject 2.

	Magnetization prepared rapid acquisition gradient echo	MP2RAGE (first inversion)	MP2RAGE (second inversion)	Diffusion tensor imaging (DTI) (Mean *b* = 0)	DTI (*b* = 700)	3D gradient and spin echo (first echo)
SNR	163.06	66.64	409.38	179.83	91.12	558.21

### Image Processing

All images were preprocessed with a customized pipeline, using a combination of software tools, including: (1) The FMRIB software library *[Oxford Centre for Functional MRI of the Brain, Oxford, UK]*, (2) Medical Image Processing, Analysis, and Visualization (MIPAV) *[Centre for Information Technology, NIH, Bethesda, MD, USA]*, (3) MATLAB *[The MathWorks Inc., Natick, MA, USA]*, (4) Statistical Parametric Mapping (SPM12) *[Wellcome Trust Centre for Neuroimaging, London, UK]*, and (5) MRIStudio *[Johns Hopkins University (JHU), Baltimore, MD, USA]*. Please note that detailed, step-by-step instructions describing how the images were processed are available in the Supplementary Material.

**Figure 1:**
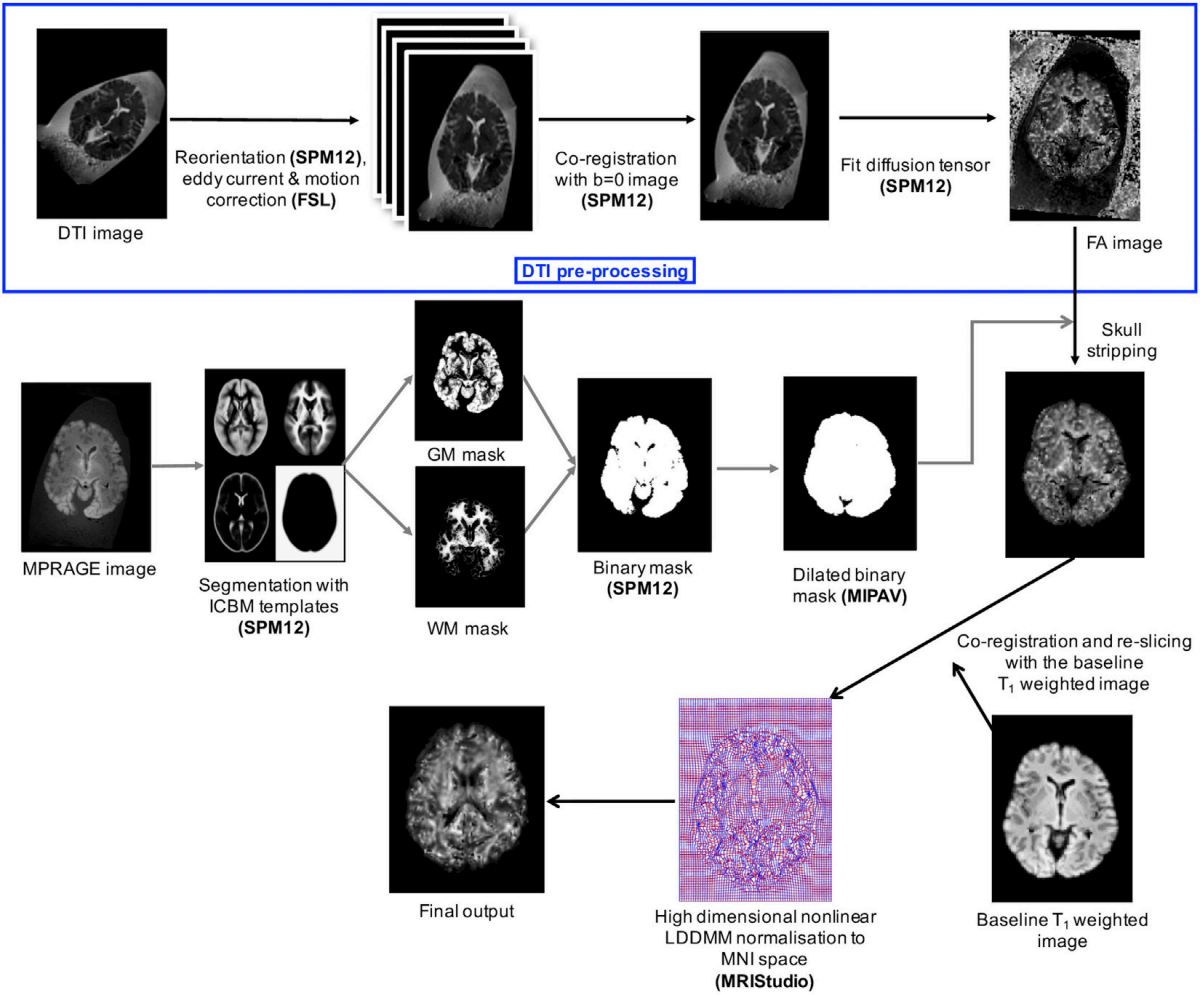
The multistage image processing pipeline used for *ex vivo* postmortem brain MRI.

Figure 1 shows the total image preprocessing pipeline. Briefly, the raw DICOM images from the scanner were converted to NIFTI format and manually reoriented to the anterior commissure–posterior commissure plane using SPM12 to facilitate subsequent automated co-registration across time points and image modalities. To get rid of unnecessary appearance of the container and formalin in the images, we used the “old segmentation” (36) tool in SPM12 and created binary GM and WM masks from the T_1_-weighted images. These binary masks were dilated in MIPAV using a 5-mm^3^ kernel before they were used for skull-stripping. All axial DTI images were eddy current corrected and motion corrected before generating FA and MD maps using the Fit Diffusion Tensor tool (37, 38) within SPM12. T_1_-relaxation maps were automatically calculated on the MRI scanner console—through the Siemens Image Reconstruction Environment—using the MP2RAGE sequence. T_2_-relaxation maps were calculated offline from multi-component multi-echo T_2_-relaxation data on a voxel-by-voxel basis using stimulated echo compensation [i.e., by taking the signal contribution from stimulated and indirect echoes arising from imperfect radiofrequency (RF) refocusing pulses] (11, 39). This method was based on the extended phase graph algorithm (40), and the RF pulse shapes and sequence timing were used to fit complete spin response. The MWF maps were calculated from the 3D GRASE sequence using multi-exponential T_2_ fitting for different water compartments while compensating for stimulated and indirect echoes due to imperfect refocusing pulses (14, 40, 41). The MWF within each voxel was obtained from the geometric mean of the T_2_ distribution from myelin water (~10–40 ms) divided by the geometric mean of the T_2_ distribution due to intra- and extra-cellular water (~100–200 ms) plus the myelin water (~10–40 ms), as previously described (35).

In order to spatially normalize the data from both subjects, all time points, and all contrasts, the T_1_-weighted data of the first four time points from each subject were spatially normalized in MRI Studio to the JHU T_1_ brain template in Montreal Neurological Institute (MNI) space (42). This was accomplished using a two-stage warping procedure consisting of a 12-parameter affine (linear) transformation, followed by high-dimensional, non-linear normalization with the large deformation diffeomorphic metric mapping (LDDMM) algorithm (43) with cascading alpha values of 0.01, 0.05, and 0.002 (44). Later, the respective combined transformation matrices (i.e., linear affine and non-linear LDDMM) were applied to the T_1_-weighted image and the rest of the co-registered images (i.e., FA, MD, T_1_, T_2_, and MWF maps) from each time point. Since the T_1_-weighted image contrast was reduced and the shape of the brain was presumed to be constant (due to the fixation beyond 120 h) during time points 5–13, the normalization parameters from time point 4 were applied to subsequent time points after co-registering the native-space images in SPM12.

**Figure 2:**
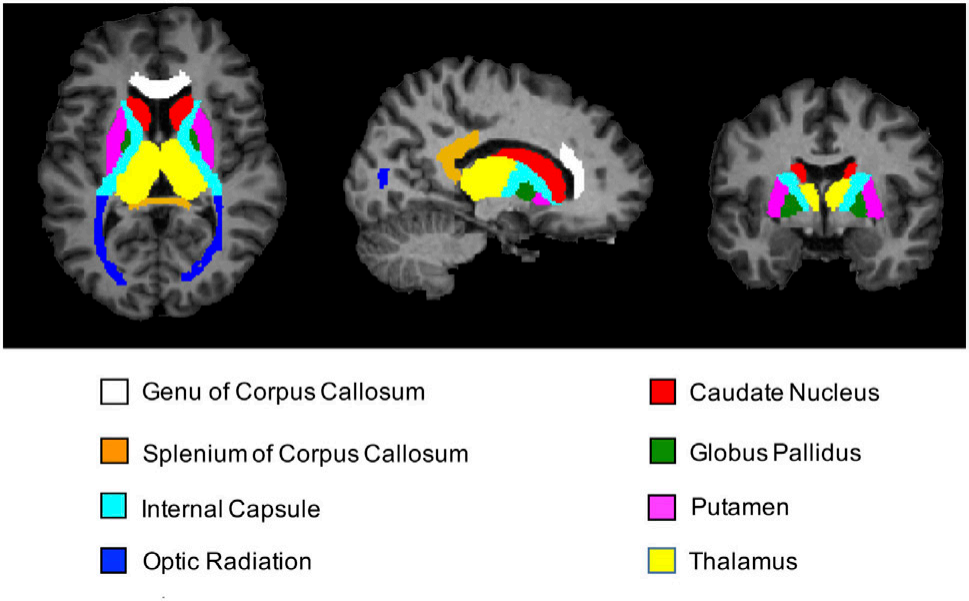
T_1_-weighted magnetization prepared rapid acquisition gradient echo images (axial, sagittal, and coronal views) showing regions-of-interest (ROIs) extracted from the JHU_MNI_SS brain atlas. Colored regions highlight the locations of all four deep white matter structures (genu of corpus callosum, splenium of corpus callosum, internal capsule, and optic radiation) and all four deep gray matter structures (caudate nucleus, putamen, globus pallidus, and thalamus) chosen for subsequent ROI analyses.

After all of the images were normalized, SPM12 was then used to map the spatiotemporal changes due to formalin fixation—on a voxel-wise level throughout the entire brain—by subtracting the baseline FA, MD, T_1_-relaxometry, T_2_-relaxometry, and MWF maps from each of the subsequent time points. Region-of-interest (ROI) analyses were performed using ROIEditor and 3D ROIs chosen from the JHU_MNI_SS (“Eve”) atlas in four deep WM and four subcortical GM structures, including genu of the corpus callosum (GCC), splenium of the corpus callosum (SCC), optic radiation (OR), internal capsule (IC), putamen (Put), thalamus (TH), globus pallidus (GP), and caudate nucleus (CN). Figure 2 shows the position of these ROIs in axial, sagittal, and coronal views. For bilateral ROIs, corresponding FA, MD, T_1_, T_2_, and MWF values for each subject were averaged across hemispheres.

### Statistical Analyses

All data were analyzed using SPSS 23.0 *[International Business Machines Corporation, Armonk, NY, USA]*. Spearman rank-correlation coefficient (*r_s_*) was used to assess correlations between MRI measures and fixation time. Two-tailed tests were performed, and only ROIs showing Bonferroni corrected *p*-values less than 0.05 (i.e., *p* < 0.00625, corrected for multiple comparisons across brain regions) were considered to be statistically significant.

### Histological Validation of WM Integrity

In order to examine tissue autolysis and degradation, and in particular, to confirm that myelin was not visibly compromised/degenerated throughout the time-course of each experiment, deep WM tissue sections were obtained from each brain specimen following the last MRI scan (i.e., after 1,032 h of formalin fixation). Specifically, tissue sections with a thickness of 5 µm were obtained from deep WM regions, stained with Eriochrome Cyanine R (Sigma-Aldrich Product ID: 1031640025; also known as Solochrome Cyanine R or Chromoxane Cyanine R), and photographed at 400× magnification using an Olympus BX53 microscope with CellSens imaging software (Olympus Life Science, Tokyo, Japan). With this preparation, myelin is selectively stained blue (45, 46),^2^ thereby allowing myelinated axons remaining at the conclusion of the experiment to be visualized.

## Results

**Figure 3:**
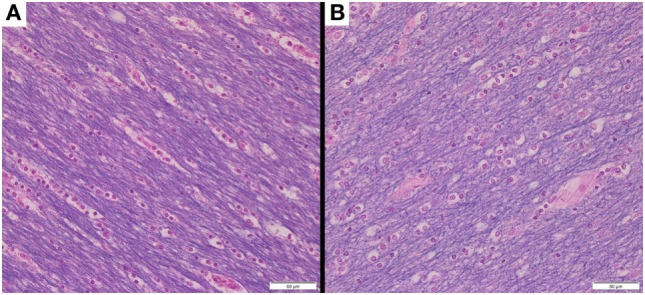
Following the conclusion of our MRI experiments, brain tissue sections were obtained from deep white matter regions of **(A)** Subject 1 and **(B)** Subject 2. Each tissue section was stained with Eriochrome Cyanine R and photographed at 400× magnification (note: 50-µm scale bar in bottom right of each panel), suggesting that the tissue in general (all colors) and myelin in particular (blue) were reasonably well-preserved throughout the 1,032 h experimental time-course (during which all MRI measurements were acquired).

It is worth noting that Eriochrome Cyanine-stained tissue sections obtained from each brain after the final, 1,032 h MRI scans appear to show: (1) little, if any, evidence of generalized autolysis and (2) large numbers of myelinated axons within the deep WM (Figure 3). The histology data, therefore, suggest that both the tissue (in general) and myelin (in particular) were reasonably well-preserved—or at least not markedly degenerated—during the course of our MRI experiments.

**Figure 4:**
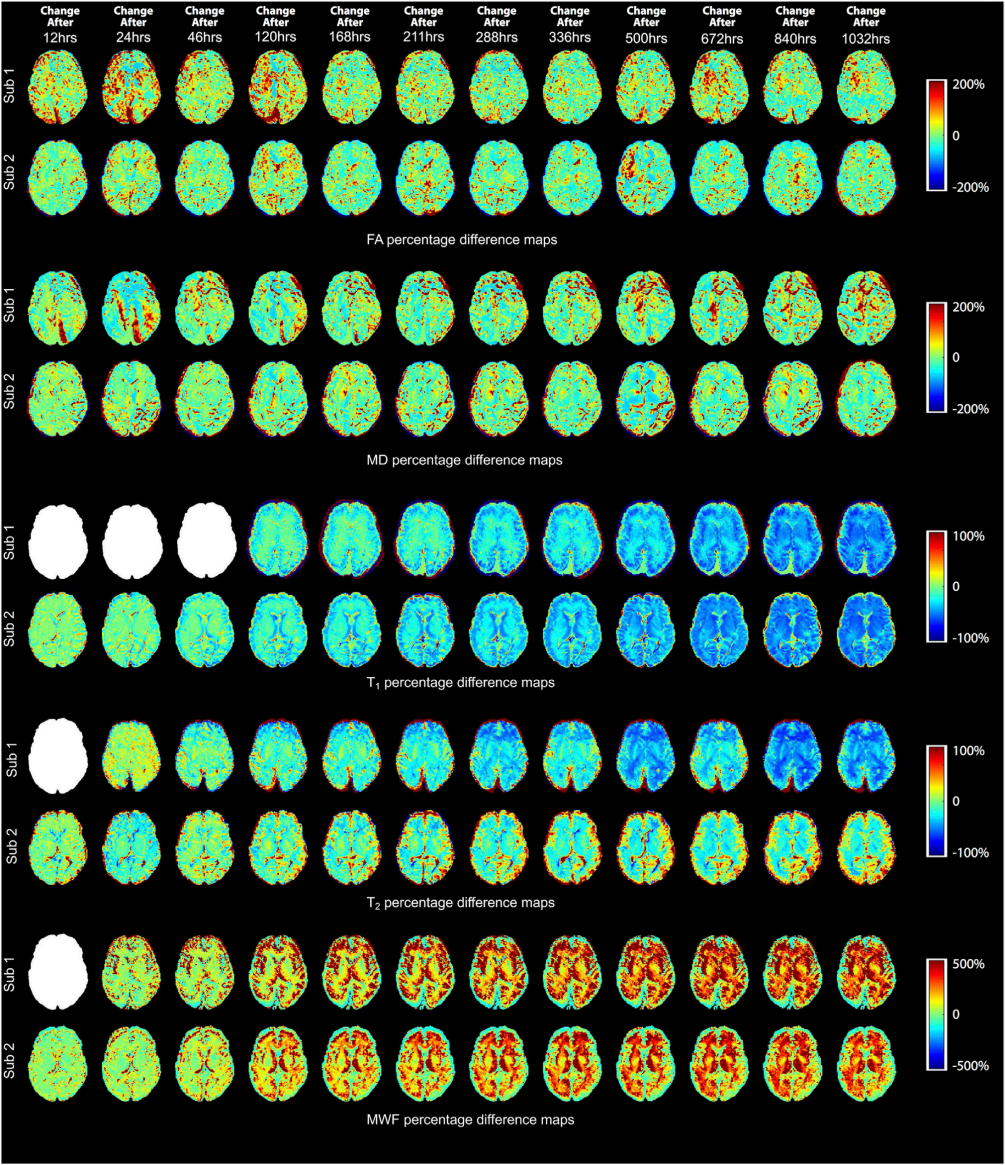
Percentage difference maps illustrating spatiotemporal changes of fractional anisotropy (FA), mean diffusivity (MD), T_1_, T_2_, and myelin water fraction (MWF) measurements at 12, 24, 46, 120, 168, 211, 288, 336, 500, 672, 840, and 1,032 h after initial fixation relative to the initial baseline measurement (i.e., 0 h in most cases). However, due to image artifacts at 0 h, the T_2_ and MWF maps of Subject 1 were compared to the images obtained at 12 h (i.e., corresponding to time point 2 for most other measurements); and due to a MP2RAGE licensing issue, the first (baseline) T_1_-relaxometry scan for Subject 1 was acquired after 46 h of formalin fixation (i.e., corresponding to time point 4 for most other measurements). Blank (white) images represent the missing difference maps due to these issues.

**Figure 5:**
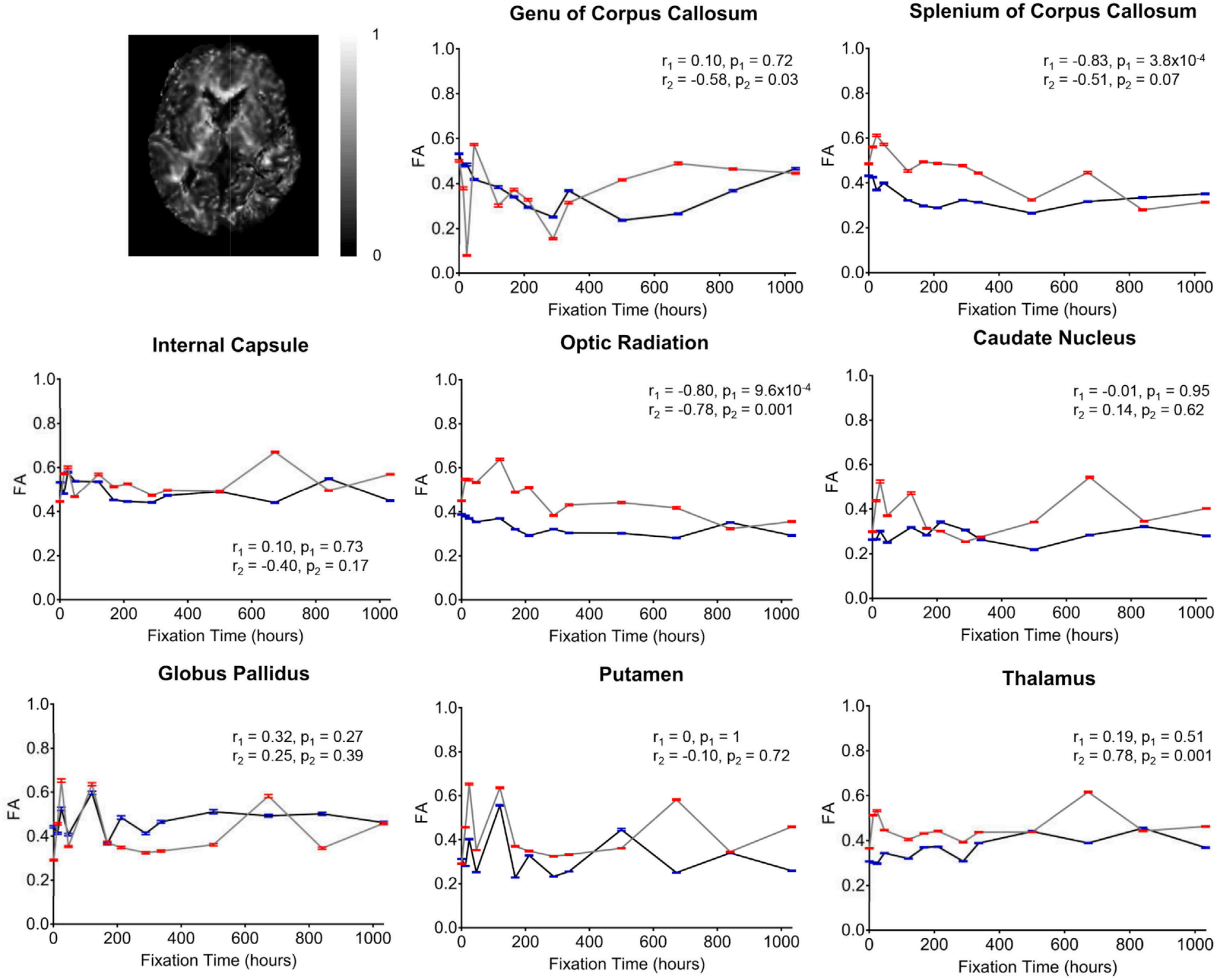
Plots of fractional anisotropy (FA) vs. formalin fixation time are shown for eight structures (genu of corpus callosum, splenium of corpus callosum, internal capsule, optic radiation, caudate nucleus, globus pallidus, putamen, and thalamus). The *x*-axis shows the time in hours, where *x* = 0 is the time of first formalin immersion. The *y*-axis shows the FA values in each structure. The lines with red and blue error bars (95% CIs) represent measurements from Subject 1 and Subject 2, respectively. Plots show FA changes in each structure throughout the fixation. An example FA map from Subject 2 has been shown in the top left.

**Figure 6:**
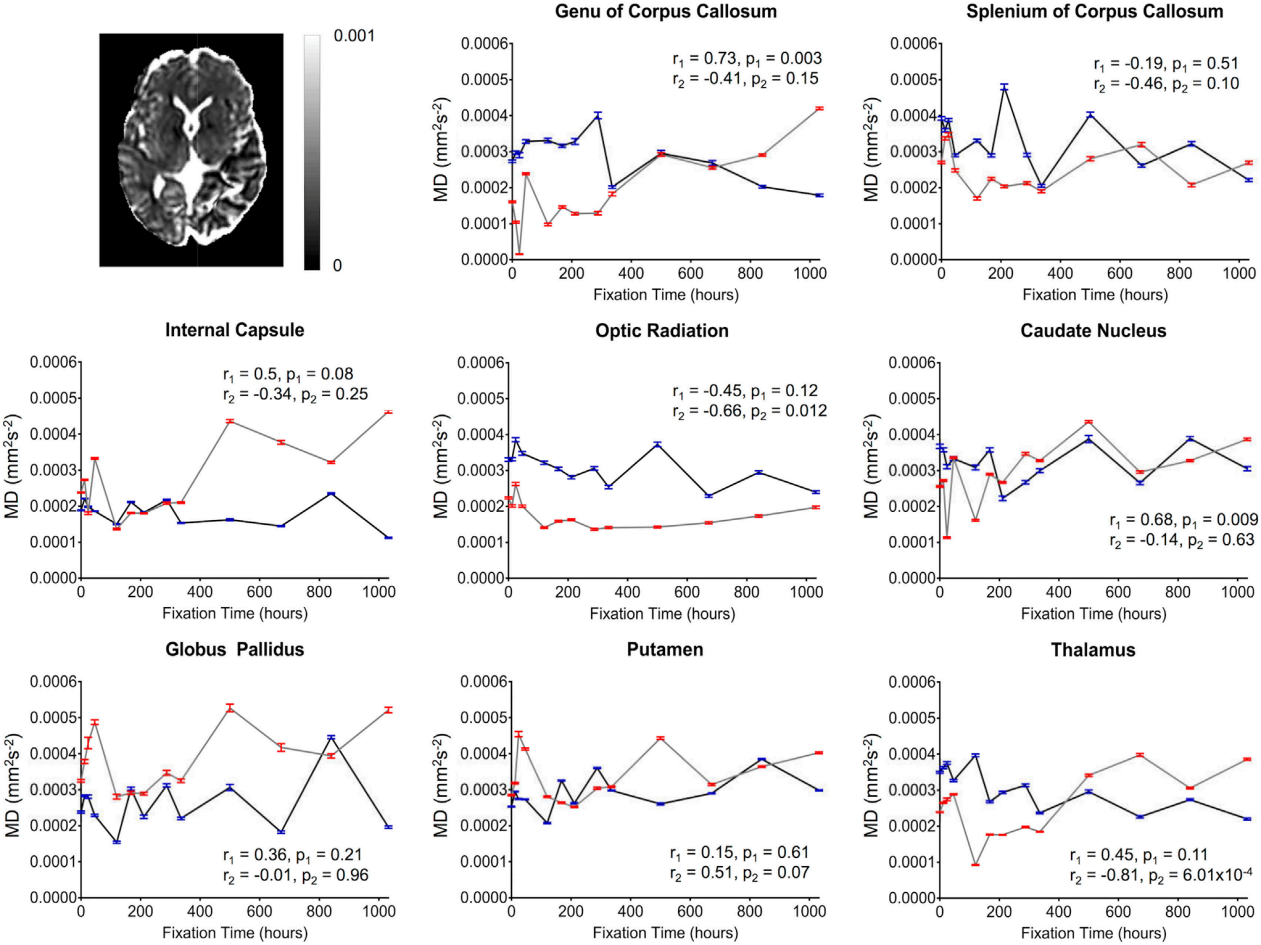
Plots of mean diffusivity (MD) vs. formalin fixation time are shown for eight structures (genu of corpus callosum, splenium of corpus callosum, internal capsule, optic radiation, caudate nucleus, globus pallidus, putamen, and thalamus). The *x*-axis shows the time in hours, where *x* = 0 is the time of first formalin immersion. The *y*-axis shows the MD values in each structure. The lines with red and blue error bars (95% CIs) represent measurements from Subject 1 and Subject 2, respectively. Plots show MD changes in each structure throughout the fixation. An example MD map from Subject 2 has been shown in the top left.

**Figure 7:**
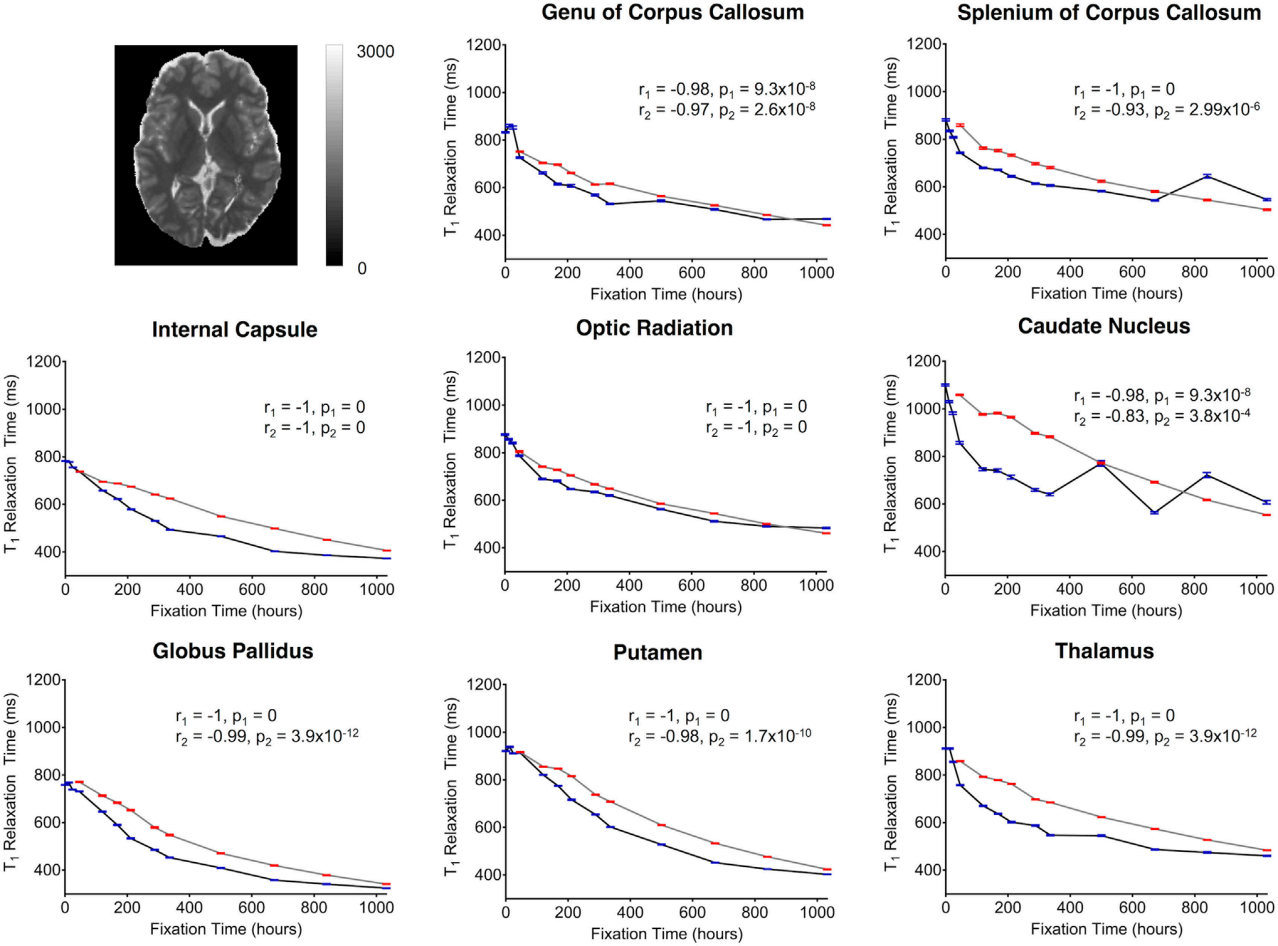
Plots of T_1_-relaxation time vs. formalin fixation time are shown for eight structures (genu of corpus callosum, splenium of corpus callosum, internal capsule, optic radiation, caudate nucleus, globus pallidus, putamen, and thalamus). The *x*-axis shows the time in hours, where *x* = 0 is the time of first formalin immersion. The *y*-axis shows the T_1_ values in each structure. The lines with red and blue error bars (95% CIs) represent measurements from Subject 1 and Subject 2, respectively. Plots show T_1_ changes in each structure throughout the fixation. An example T_1_ map from Subject 2 has been shown in the top left.

**Figure 8:**
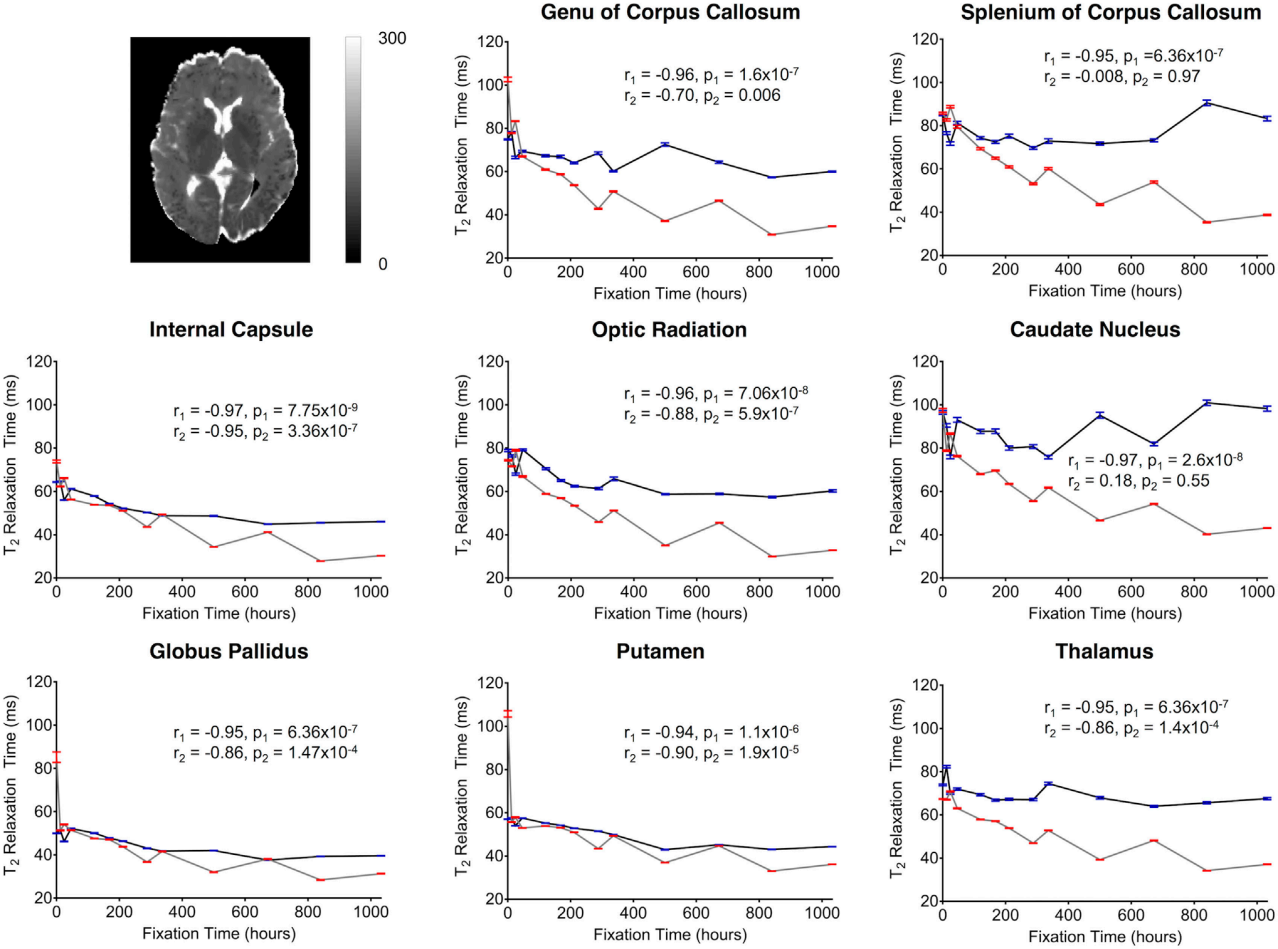
Plots of T_2_-relaxation time vs. formalin fixation time are shown for eight structures (genu of corpus callosum, splenium of corpus callosum, internal capsule, optic radiation, caudate nucleus, globus pallidus, putamen, and thalamus). The *x*-axis shows the time in hours, where *x* = 0 is the time of first formalin immersion. The *y*-axis shows the T_2_ values in each structure. The lines with red and blue error bars (95% CIs) represent measurements from Subject 1 and Subject 2, respectively. Plots show T_2_ changes in each structure throughout the fixation. An example T_2_ map from Subject 2 has been shown in the top left.

**Figure 9:**
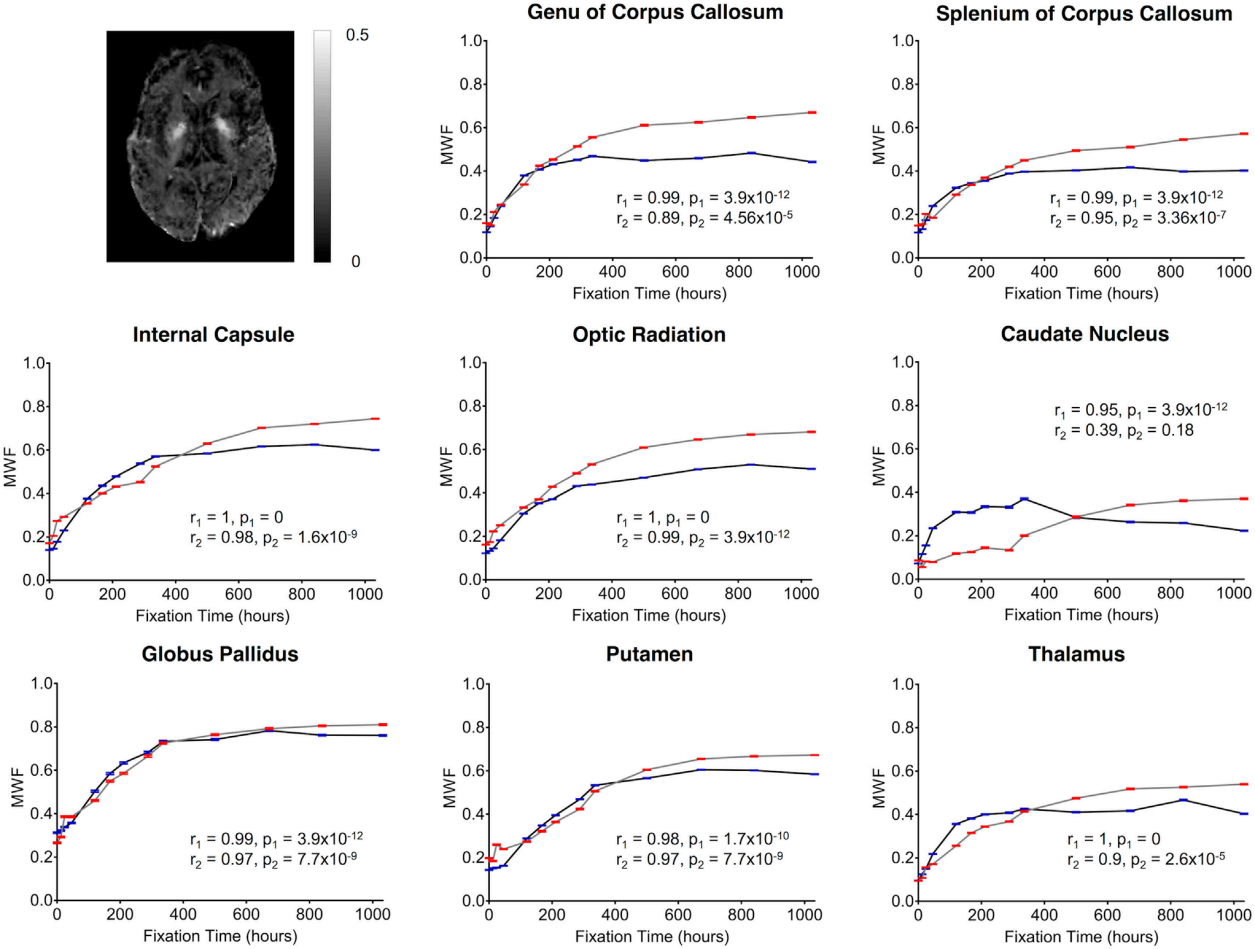
Plots of MWF vs. formalin fixation time are shown for eight structures (genu of corpus callosum, splenium of corpus callosum, internal capsule, optic radiation, caudate nucleus, globus pallidus, putamen, and thalamus). The *x*-axis shows the time in hours, where *x* = 0 is the time of first formalin immersion. The *y*-axis shows the MWF values in each structure. The lines with red and blue error bars (95% CIs) represent measurements from Subject 1 and Subject 2, respectively. Plots show MWF changes in each structure throughout the fixation. An example MWF map from Subject 2 has been shown in the top left.

The percent change maps for FA, MD, T_1_, T_2_, and MWF at different time points relative to the baseline (i.e., first time point) are shown in Figure 4 to visualize the spatiotemporal variations of each quantitative MRI measure across the entire brain. Then, to better understand some of the ROI-specific alterations, we plotted each of the MRI measures (FA, MD, T_1_, T_2_, and MWF) over fixation time (in hours) for eight deep WM and GM structures, as shown in Figures 5–9 (which also show examples of FA, MD, T_1_, T_2_, and MWF maps). Corresponding correlation coefficients and *p*-values are presented in Table 3.

**Table 3:** Subject-wise Spearman correlations between formalin fixation time and quantitative MRI values.^a^

			Genu of the CC	Splenium of the CC	Internal capsule	Optic radiation	Caudate nucleus	Globus pallidus	Putamen	Thalamus
Fractional anisotropy	Subject 1	*r_s_*	0.1	**−0.83**	0.1	**−0.8**	−0.01	0.32	0	0.19
		*p*-value	0.72	**<0.001**	0.73	**<0.001**	0.95	0.27	1	0.51
	Subject 2	*r_s_*	−0.58	−0.51	−0.4	**−0.78**	0.14	0.25	−0.1	**0.78**
		*p*-value	0.03	0.07	0.17	**0.001**	0.62	0.39	0.72	**0.001**

Mean diffusivity	Subject 1	*r_s_*	**0.73**	−0.19	0.5	−0.45	0.68	0.36	0.15	0.45
		*p*-value	**0.003**	0.51	0.08	0.12	0.009	0.21	0.61	0.11
	Subject 2	*r_s_*	−0.41	−0.46	−0.34	−0.66	−0.14	−0.01	0.51	**−0.81**
		*p*-value	0.15	0.10	0.25	0.012	0.63	0.96	0.07	**<0.001**

T_1_	Subject 1	*r_s_*	**−0.98**	**−1**	**−1**	**−1**	**−0.98**	**−1**	**−1**	**−1**
		*p*-value	**<10^−7^**	**0**	**0**	**0**	**<10^−7^**	**0**	**0**	**0**
	Subject 2	*r_s_*	**−0.97**	**−0.93**	**−1**	**−1**	**−0.83**	**−0.99**	**−0.98**	**−0.99**
		*p*-value	**<10^−7^**	**<10^−5^**	**0**	**0**	**<0.001**	**<10^−11^**	**<10^−9^**	**<10^−11^**

T_2_	Subject 1	*r_s_*	**−0.96**	**−0.95**	**−0.97**	**−0.96**	**−0.97**	**−0.95**	**−0.94**	**−0.95**
		*p*-value	**<10^−6^**	**<10^−6^**	**<10^−8^**	**<10^−7^**	**<10^−7^**	**<10^−6^**	**<10^−5^**	**<10^−6^**
	Subject 2	*r_s_*	**−0.70**	−0.008	**−0.95**	**−0.88**	0.18	**−0.86**	**−0.90**	**−0.86**
		*p*-value	**0.006**	0.97	**<10^−6^**	**<10^−6^**	0.55	**<0.001**	**<0.0001**	**<0.001**

MWF	Subject 1	*r_s_*	**0.99**	**0.99**	**1**	**1**	**0.95**	**0.99**	**0.98**	**1**
		*p*-value	**<10^−11^**	**<10^−11^**	**0**	**0**	**<10^−11^**	**<10^−11^**	**<10^−9^**	**0**
	Subject 2	*r_s_*	0.89	**0.95**	**0.98**	**0.99**	0.39	**0.97**	**0.97**	**0.9**
		*p*-value	**<10^−4^**	**<10^−6^**	**<10^−8^**	**<10^−11^**	0.18	**<10^−8^**	**<10^−8^**	**<0.0001**

*^a^Correlations with *p* < 0.00625 (Bonferroni corrected) are highlighted with bold font. CC, corpus callosum*.

Overall, the diffusion imaging measurements appeared to be noisier and (perhaps in part because of this) less affected by formalin fixation than either relaxometry or myelin water measurements. No systematic differences were observed in the whole-brain difference (percent change) maps (Figure 4), and the ROI analyses of FA values (Figure 5) revealed no significant differences in the majority of structures (i.e., *p* > 0.05 in six out of eight structures). In both subjects, OR (*p* = 9.6 × 10^−4^ and *p* = 0.001, respectively) showed gradual, albeit statistically significant decreases, and Subject 1 and Subject 2 showed statistically significant increases in the SCC (*p* = 3.8 × 10^−4^) and TH (*p* = 0.001) respectively. Similarly, no significant changes in MD values (Figure 6) were observed in seven of eight structures for both subjects. Subject 1 showed significant positive correlations in the GCC (*p* = 0.003), while Subject 2 showed strong negative correlations in the TH (*p* = 6.1 × 10^−4^). However, the baseline (i.e., first time point) FA values in WM regions were generally higher than those in GM regions, and the baseline MD values in WM regions were generally lower than those in GM regions, as expected (6, 47).

For both subjects, the relaxation times (T_1_ and T_2_) were significantly affected by formalin fixation. Clear spatiotemporal changes were observed across the entire brain (Figure 4), and ROI analyses confirmed that: (1) T_1_-relaxation times were generally reduced by 30–60% at the final time point compared to baseline, and (2) these reductions were statistically significant in both WM and GM structures (*p* ≤ 10^−4^ in all cases) (Figure 7). Similarly, T_2_-relaxation times appeared to be reduced by progressive formalin fixation, with most regions showing significant negative correlations (Figure 8). However, T_2_-relaxation time measurements were not significantly changed in the SCC (*p* = 0.97) or CN (*p* = 0.55) for Subject 2.

Finally, MWF measurements showed clear fixation-related changes across the entire brain (Figure 4), and the apparent MWF was significantly increased (*p* < 0.05) in all eight WM and GM regions for Subject 1, and seven out of eight regions for Subject 2 (Figure 9). However, although the MWF in the CN of Subject 2 did not produce a significant overall Spearman rank correlation (*p* = 0.18, due to the decreased later time points, relative to the middle time points), it should be noted that all of the MWF estimates beyond the third time point (i.e., 24 h) were still at least twice as large as the initial baseline value.

Although there were between-subject differences in absolute MD, FA, T_1_-relaxation, T_2_-relaxation, and MWF measurements (Figures 5–9), it should be noted that formalin-related changes exhibited remarkably similar trends in both brain specimens. As is clearly illustrated in Figure 4 (and can also be seen in Figures 5–9), the diffusion measurements (i.e., MD and FA) did not show significant overall trends in either case (Figures 5 and 6), T_1_-relaxation times were decreased in both cases (Figure 7), T_2_-relaxation times were decreased in both cases (Figure 8), and MWF estimates were increased in both cases (Figure 9). The largest systematic difference between subjects is that the reductions in T_2_-relaxation times were consistently larger and more significant for Subject 1 compared to Subject 2 (apparent in Figure 4 and shown in detail in Figure 8).

## Discussion

We have demonstrated the time-dependent effects of formalin fixation on various quantitative MRI measurements (i.e., FA, MD, T_1_, T_2_, and MWF) throughout whole human brain samples and have characterized these changes in several WM and GM tissue structures. We found that formalin fixation did not systematically effect diffusion measurements (FA and MD values) as significantly as other quantitative measures: namely, T_1_-relaxation times, T_2_-relaxation times and MWF estimates, which were progressively altered by tissue fixation.

Although the diffusion-weighted FA and MD values in the current study were not significantly correlated with formalin fixation time, we cannot conclude from these measurements that fixation did not affect diffusion. Rather, the fact that large signal fluctuations were observed between consecutive scans (Figures 5 and 6) suggests that the variability was simply dominated by other factors during (1) preprocessing (e.g., co-registration, spatial normalization, etc.) or (2) image acquisition. However, given that the same preprocessing pipeline and spatial normalization parameters were used for the diffusion data and the rest of the contrast maps (which did not show similar fluctuations), we suspect that the underlying cause of these instabilities are more likely related to the image acquisition parameters and the reduced contrast-to-noise ratio (CNR) associated with *ex vivo* diffusion imaging. For example, the ~15°C difference between room temperature (~22°C) and body temperature (~37°C) means that the actual amount of water diffusion is reduced compared to *in vivo* conditions (all else being equal), which is why we sought to use stronger diffusion-encoding than some earlier *ex vivo* diffusion imaging studies (23). It is worth noting that previous studies comparing diffusion characteristics in fixed and unfixed rat optic nerves scanned at physiological temperature (37°C) found that tissue fixation lead to significantly reduced axial diffusivity (parallel to WM tracts, which would translate to both lower FA and lower MD values, all else being equal) and increased radial diffusivity (perpendicular to WM tracts, which would translate to lower FA and higher MD values, all else being equal) (48). These results are consistent with our findings in the OR, where: (1) FA values were significantly reduced (*p* < 0.001) in both specimens, but (2) changes in MD values over time were either not significant (Subject 1; *p* = 0.12) or less significant (Subject 2; *p* = 0.012).

However, one factor that we did not adequately account for was that as T_2_-relaxation times are decreased with progressive fixation (Figure 8), ostensibly “diffusion weighted” images become increasingly dominated by T_2_-weighting. As a result, using conventional *in vivo* diffusion-encoding parameters—e.g., *b* = 700 s/mm^2^ and relatively long echo times (TE > 70 ms), as in the current experiment—inherently confounds the effects of diffusion and T_2_-relaxation changes. Nonetheless, despite these confounds and the fact that higher amounts of diffusion-sensitivity also comes at the expense of SNR (49), future *ex vivo* diffusion imaging studies could potentially reduce these confounds and improve image SNR by using different data acquisition parameters (e.g., stronger diffusion gradients, shorter TEs, higher field strengths, longer scan times, etc.) that were not possible in the current experiment.^3^ Nonetheless, a strong FA vs. fixation time correlation (*r*_1_ = −0.8, *p*_1_ = 9.6 × 10^−4^; *r*_2_ = −0.78, *p*_2_ = 0.001) was observed in the OR for both subjects. This fiber bundle has higher myelin density than the surrounding tissue (50), which may partially explain the higher directional anisotropy (and higher CNR) compared to other structures.

Consistent with other previous studies (13, 15, 17, 27, 51, 52), T_1_-relaxation times decreased with time of formalin fixation for all structures. These T_1_ changes may be a result of the viscosity of the fixation media, radius, spin-spin distance of molecules and protein cross-linking as described by an earlier study (53). Compared with T_1_ changes, the T_2_ shortening appeared to be slower, but these changes were still highly significant, and were similar to earlier studies (8, 22, 23). Though the mechanisms are not known, increased tissue rigidity due to formalin-induced protein cross-linking may play a role in T_2_ shortening as well. One previous study has mentioned the possibility of interactions between myelin lipids and water molecules inside the tissue compartments (54), while another study suggested that an altered “exchange diffusion rate” between free and bound water molecules may play a role (26). Additionally, tissue dehydration (22, 28) and replacement of formalin solution at regular intervals are thought to have T_1_ and T_2_ shortening effects (55).

In stark contrast to relaxometry measures, the MWF values increased rapidly and all regions showed highly significant positive correlations with fixation time—with the exception of the GCC and CN for Subject 2. To the best of our knowledge, this is the first study to investigate relationships between MWF measurements and tissue fixation; and, although our data do not speak directly to the exact mechanisms underlying these MWF changes, we predict that there are two simultaneous effects that may be contributing. First, there is likely an increase in the volume of “myelin water” (i.e., increasing the numerator in the MWF) due to oxygen and glucose deprivation, which have previously been linked to changes in myelin layer densities in brain tissue (56). If the myelin is less tightly bound, there could be an increase in myelin water, without an increase in myelin *per se*. Moreover, we speculate that tissue dehydration is again likely to play a key role, where a reduction of intra- and extra-cellular water (17) in brain tissue—most likely due to the chemical reaction between water and refilled formalin (2)—would artificially inflate apparent MWF values by reducing the denominator (i.e., myelin plus intra/extra-cellular water) in the MWF equation. Our T_2_ shortening values arising from the intra/extra-cellular water components tend to support this notion, although further investigation is required to support this theory and to understand the exact cause(s) for these MRI changes. Although it was not possible to investigate in our current study, future experiments may be able to test this hypothesis by, for example, taking measurements from a formalin-fixed brain and then washing out the fixative and rehydrating the brain tissue in order to determine whether MWF values are decreased back to *in vivo* (or early *ex vivo*) levels. Nonetheless, our findings clearly indicate that even small differences in fixation time between tissue samples could have large effects on the measurements, and that researchers must exercise extreme caution when comparing *in vivo* MWF values with *ex vivo* values (or MWF values acquired at different fixation times).

### Study Limitations

Despite our best efforts to optimize the experimental design, acquisition, and analysis, there are a few noteworthy limitations in this study (in addition to the challenges mentioned above regarding the *ex vivo* diffusion measurements).

It should be noted that tissue sectioning and Eriochrome Cyanine staining was only performed after 1,032 h in the current experiments to facilitate the longitudinal MRI components of the study and verify (to the extent possible) that tissues were not structurally compromised throughout the relatively long experimental time-course. Under normal circumstances, sectioning and staining should be performed much sooner (e.g., typically between 2 and 4 weeks according to standard pathology practices) in order to avoid protein cross-linking and other potential fixation-related immunohistochemistry artifacts.

Due to ethical and logistical constraints (e.g., BREB approval, patient consent, availability of neurologically healthy adult brain specimens, autopsy scheduling, and availability of MRI scanner time) our sample size was small (*n* = 2) and PMIs were not as short or consistent as desired (in order to inspect the immediate postmortem changes and minimize possible tissue degradation prior to fixation). Therefore, although both specimens were refrigerated almost immediately after patient death, differences in PMI (i.e., 33 vs. 55 h) and any associated tissue degradation prior to fixation may have had an effect on the results, as PMI is known to affect MRI properties (57–59). Partial volume effects due to tissue segmentation, subcortical structures adjacent to formalin, and imperfect spatial normalizations to the MNI template may have affected quantitative precision in MRI measures. Furthermore, despite the fact that other studies suggest that tissue fixation continues to change MRI properties up to 1,142 h (51) or even a year (52), we were unable to evaluate the MRI changes beyond 1,032 h in our study because the samples had to undergo neuropathological examinations for diagnostic purposes at that time (in accordance with standard neuropathology practices at our site, and in accordance with our BREB approval).

Another limitation was that, in order to reduce the overall scan time for each session (given that there were 26 sessions), T_1_-relaxometry was performed using an MP2RAGE sequence with parameters optimized for *in vivo* and early *ex vivo* T_1_-relaxation values. However, because the MP2RAGE method only uses two inversion times—thereby enabling fast data acquisition compared to conventional T_1_-relaxometry methods based on multiple inversion pulses—there are certain inherent assumptions (e.g., perfect inversion efficiency, etc.) that are violated as T_1_-relaxation times stray farther from the optimized range of values. As a result, the accuracy of our MP2RAGE-based T_1_ measurements may have been decreased at later fixation times (as the values became increasingly short). However, the changes observed between the first and last measurements, although statistically significant, were still on the same order of magnitude (e.g., starting ~900 ms and decreasing to ~500 ms); and perhaps even more importantly, we reported and based our conclusions on Spearman rank correlations (as opposed to linear Pearson correlations), which do not depend on the absolute values of the measurements, but rather the overall trends (i.e., monotonic increases or decreases) over time.

T_2_-relaxometry was performed using multi-echo 3D GRASE images from the MWI sequence with a relatively short TR: thereby introducing more T_1_-weighting than traditional T_2_-relaxometry methods. However, we did not have enough time in each session to acquire separate spin echo based T_2_-relaxometry data, and since the amount of T_1_-weighting was constant across samples and time points, these effects are presumed to be minimal. Nonetheless, since significant T_1_-relaxation changes were identified, there may have been some interaction between T_1_ and T_2_ effects in the T_2_-relaxation time measurements.

At least one previous study noted that the position of the tissue in formalin should be taken into consideration while characterizing longitudinal effects of fixation (8); however, our plastic container was filled by formalin so that the whole cortical surface had uniform contact with formalin for the entire study duration. Furthermore, our whole-brain maps of the spatiotemporal changes suggest that tissue fixation occurred evenly, as the formalin perfused deeper into the tissue over time, eventually penetrating deep into each of the brain specimens.

## Conclusion

In this study, we characterized the time-dependent effects of formalin fixation on FA, MD, T_1_-relaxation, T_2_-relaxation, and MWF values in whole postmortem human brains. The T_1_, T_2_, and MWF changes indicate that formalin gradually diffuses inward from the cortical surface, and tissue fixation continues to affect MR properties until (and likely beyond) our maximum fixation time of 1,032 h. Given the significant and almost immediate effects of formalin fixation on *ex vivo* relaxometry (T_1_ and T_2_) mapping and MWI measurements, our results indicate that future *ex vivo* relaxometry and MWI studies should consider scanning: (1) *in situ* immediately following death (if feasible), or (2) *ex vivo* as soon as possible following both death (i.e., to minimize tissue degradation) and immersion in formalin (i.e., to avoid fixative-related differences)—preferably within the first 120 h of fixation (if possible). Our results suggest that even scanning different brains at a consistent post-fixation interval—especially between 120 and 1,032 h following initiation of formalin fixation—may lead to significantly different absolute measurements between samples, which could in turn lead to erroneous conclusions. On the other hand, *ex vivo* diffusion imaging measures (FA and MD) appeared to be more affected by factors (e.g., temperature, etc.) other than formalin, even after prolonged fixation.

Finally, considering the relatively low cost of the fixation and imaging protocols employed in the current study, these practices should be highly adoptable not only for future research applications but also in forensic autopsy practice, since it provides high quality images with a number of different tissue contrasts within a clinically acceptable time-frame. In this regard, *ex vivo* brain imaging at early time points could provide quantitative MRI data that may be useful in its own right, and also potentially guide subsequent brain sectioning and immunohistochemistry studies, which are typically performed at later postmortem intervals (e.g., between 2 and 4 weeks).

## Ethics Statement

This study was conducted with prior approval from the University of Manitoba Biomedical Research Ethics Board (BREB).

## Author Contributions

AS, KM, and CF acquired the data; AS, MU, KM, and CF were involved in data analysis and interpretation, and wrote the manuscript.

## Conflict of Interest Statement

The authors declare that the research was conducted in the absence of any commercial or financial relationships that could be construed as a potential conflict of interest.

## Footnotes

^1^Of the two T1-weighted images acquired using the MP2RAGE sequence, the ones with the longer inversion times (2,500 ms) and higher flip angles (5°) were also used for subsequent image processing steps, including co-registration and spatial normalization, etc.

^2^However, because Eriochrome Cyanine staining cannot differentiate between intact myelin vs. myelin fragments (which would require the use of more sophisticated methods such as electron microscopy), the stained images reflect the location and relative amount of total (aggregate) myelin.

^3^It is worth noting that stronger diffusion-encoding gradients, allowing higher *b*-values and/or shorter TEs, might be particularly effective for future *ex vivo* diffusion MRI experiments, since these would maximize both SNR and diffusion CNR (all else being equal), without having to worry about neurostimulation, which places practical limits on the maximum gradient strength that can be used for *in vivo* diffusion imaging.
