# Supplementary material for: Quantitative Ex Vivo MRI Changes due to Progressive Formalin Fixation in Whole Human Brain Specimens: Longitudinal Characterization of Diffusion, Relaxometry, and Myelin Water Fraction Measurements at 3T
Source: Front Med (Lausanne). 2018 Feb 20;5:31. doi: 10.3389/fmed.2018.00031 (PMC5826187; doi:10.3389/fmed.2018.00031)
Supplement: Supplementary file 1 [file Presentation_1.PDF]

Image Processing Steps: Supplement

1. ***Dicom to NIFTI conversion***: All DICOM images were converted to NIFTI format using MATLAB 'dicm2nii' tool[1].

2. ***Checking Orientation***: After the conversion, all images were checked for correct orientation using the SPM12[2] "Display" option. The origin of the images was roughly set to the anterior commissure (AC) to ensure proper co-registration across the time points and image modalities.
   [Instructions: Display > (Open image) > (Manually set origin to AC) > Set Origin > Apply to applicable image/s]

3. ***Eddy current and motion correction***: DTI images are stretched and sheared by Eddy currents induced in the gradient coils. As a result, these distortion need to be corrected. FSL[3] "FDT Diffusion" toolbox[4] corrects for these distortions and also for head movement (motion). Since a reference image is needed, the first b = 0 image was selected.
   [Instructions: Mac Terminal > Type 'FSL' > Select 'FDT Diffusion' > Choose DTI nifti image > Leave 'Reference' to 0]

4. ***Obtaining FA and MD images***: For each time point, DTI images were preprocessed using the SPM12 Artefact Correction In Diffusion (ACID) toolbox. This does not come with the SPM package, so needs to be downloaded separately (link: http://www.diffusiontools.com/documentation/TensorFit.html). Appropriate DTI images, b-vectors and b-values (*.bvec and *.bval files created in Step 1) were required to fill in appropriate fields. Remaining options were left at their default values. This step created FA and MD maps of each time point.
   [Instructions: Batch > SPM > Tool > ACID Tollbox > Tensor Fitting > Fit Diffusion Tensor]

5. ***Image Segmentation***: To extract the brain structure from the surrounding formalin and cotton, we used the 'Old Segmentation' toolbox in SPM12 (simply called 'Segmentation' tool in SPM8). MPRAGE/MP2RAGE T1-weighted images were used as inputs, and based on the ICBM tissue probability maps (integrated in SPM), this step created binary GM and WM segments along with forward and inverse segmentation matrices of the T1-weighted images. CSF segments were not saved. All other options remained unchanged.
[Instructions: Batch > SPM > Tool > Old Segment]

6. ***Image Co-registration***: All images from each brain specimen/time-point were co-registered to their respective T1-weighted image. SPM12 "Co-register: Estimate and Re-slice" option was used for this purpose.
[Instructions: Click 'Co-register: Estimate and Re-slice' on the GUI]

7. ***Brain Extraction (Skull Stripping)***: GM and WM segments were combined (added) to create a binary mask for each time-point. To cover the missing portions of CSF inside the brain, all binary masks were dilated in MIPAV[5] using a 5x5x5-24 connected kernel. Later, all images were skull-stripped by simple multiplication of the original image and the binary mask.
[MIPAV Instructions for dilation: Open image > Algorithm > Morphological > Dilate]

8. ***Image Normalisation***: In order to keep the assessment constant across subjects and time-points, images were spatially normalized. First, each image from each time point was coregistered and non-linearly warped using the forward matrices created in Step-5 above. For this purpose, SPM "Old Normalise" tool was used (for earlier SPM versions, it is named as "Normalise").
[Instructions: Batch > SPM > Tool > Old Normalise > Old Normalise: Write]

Then, to more precisely normalise the data, the SPM-normalised T1-weighted images of the first four time points from each subject were spatially normalized in MRIStudio[6] (using the "DifffeoMap" tool) to the JHU T1-weighted brain template in MNI space. This was accomplished using a two-stage warping procedure consisting of a 12-parameter

affine (linear) transformation, followed by high-dimensional, non-linear normalization with the large deformation diffeomorphic metric mapping (LDDMM) algorithm with cascading alpha values of 0.01, 0.05 and 0.002. Later, the respective combined transformation matrices (i.e. airlinearOutput.air and Kimap.vtk) were applied to the T1-weighted image and the rest of the co-registered SPM-warped images (i.e., FA, MD, T1, T2 and MWF maps) from each time-point.

[Diffeomap Instructions:

(a) *Sending to JHU server*: Load Template (select "JHU_MNI_SS_T1_ss") > Load Subjects (select your T1-weighted image) > BFW (in the "Image" pane, select "Automatic Option" > Air Linear (in the "Transformation" pane, save the output) > Automatic Histogram Matching > Single Channel (under "Volume LDDMM" pane > Chane "Alpha" values (0.01, 0.05 and 0.002) > Submit (this will send everything to JHU server and you will receive an email after completion)

(b) *After downloading*: Load Template (select "JHU_MNI_SS_T1_ss") > Load Subjects (select your T1-weighted image) > Combine Matrices (in 'Transformation' pane, combine airlinearOutput.air and Kimap.vtk matrices that were downloaded from the server) > Load Transformation Matrix (select the combined matrix) > Save the output]

Since the T1-weighted image contrast was reduced in later scans (with longer formalin fixation) and the shape of the brain was presumed to remain constant (due to the fixation) beyond 120 hours, the normalization parameters from time point 4 were applied to subsequent time-points (i.e., time-points 5-13) after co-registering the native-space images in SPM12.

9. ***ROI Analysis***: All ROI analyses were performed using "ROI Editor" which comes in the MRIStudio package. Although this package allows for any user-defined ROIs to be loaded and evaluated, we simply used the pre-existing structural ROI database in this case. A detailed set of instructions have already been developed for this, and these can be found here: https://www.mristudio.org/wiki/user_manual/roieditor.

Software Download Links:

1. https://www.mathworks.com/matlabcentral/fileexchange/42997-dicom-to-nifti-converter--nifti-tool-and-viewer?requestedDomain=www.mathworks.com
2. https://www.nitrc.org/projects/spm
3. https://www.nitrc.org/projects/fsl
4. https://fsl.fmrib.ox.ac.uk/fsl/fslwiki/FDT
5. https://www.nitrc.org/projects/mipav
6. https://www.nitrc.org/projects/mri_studio
